# Survival of Metastatic Human Papillomavirus (HPV)-Related Head and Neck Cancer Receiving Platinum-Based Triplet Induction Chemotherapy and Relevance of Circulating Tumor HPV DNA

**DOI:** 10.7759/cureus.60547

**Published:** 2024-05-18

**Authors:** Hirotaka Eguchi, Yukinori Takenaka, Hidenori Tanaka, Motoyuki Suzuki, Masafumi Horie, Haruka Kanai, Yuji Seo, Kazuhiko Ogawa, Shinichi Yachida, Hidenori Inohara

**Affiliations:** 1 Head and Neck Surgery, Osaka University, Suita, JPN; 2 Basic Sciences, Osaka University, Suita, JPN; 3 Radiation Oncology, Osaka University, Suita, JPN; 4 Otorhinolaryngology - Head and Neck Surgery, Osaka University Graduate School of Medicine, Suita, JPN

**Keywords:** squamous cell carcinoma, metastasis, circulating tumor dna (ctdna), oropharynx cancer, human papilloma virus

## Abstract

Objectives

We aimed to examine the effectiveness of platinum-based triplet induction chemotherapy in metastatic squamous cell carcinoma of the head and neck (HNSCC) at diagnosis in terms of tumor human papillomavirus (HPV) status and the clinical relevance of circulating tumor HPV DNA (ctHPVDNA) during induction chemotherapy.

Methods

Twenty-one patients were included. ctHPVDNA was longitudinally quantified using optimized digital PCR in a subset of patients.

Results

HPV-related HNSCC patients (N=7) had a significantly better response to induction chemotherapy than HPV-unrelated HNSCC patients (N=14) (complete or partial response rate, 100% vs. 36%, *P* = 0.007). Following induction chemotherapy, more HPV-related HNSCC patients than HPV-unrelated patients received radiotherapy (86% vs. 36%, *P* = 0.06). With a median follow-up of 26 months in surviving patients, the two-year overall survival was 86% in HPV-related HNSCC patients and 43% in HPV-unrelated HNSCC patients (*P* = 0.04). In two patients, ctHPVDNA levels drastically decreased after the first cycle of induction chemotherapy but turned to continuous increase after the second cycle, suggesting the acquisition of drug resistance by the end of the second cycle. Radiographic imaging after induction chemotherapy failed to identify the drug resistance. In one patient, ctHPVDNA decreased gradually but remained detectable after induction chemotherapy despite no radiographic residual disease. ctHPVDNA became undetectable during radiotherapy.

Conclusion

HPV-related HNSCC patients with distant metastasis at diagnosis should be treated definitively. The ctHPVDNA level reflects real-time disease activity. ctHPVDNA monitoring during induction chemotherapy could help the decision-making of the therapeutic strategy.

## Introduction

Squamous cell carcinoma of the head and neck (HNSCC) is etiologically classified into two types: human papillomavirus (HPV)-related HNSCC and HPV-unrelated HNSCC. HPV-related HNSCC is caused by infection with high-risk HPV, especially HPV type16 (HPV16), while HPV-unrelated HNSCC is caused by tobacco and alcohol abuse. Squamous cell carcinoma of the oropharynx (OPSCC) dominates HPV-related HNSCC [1]. p16 overexpression on immunostaining is a surrogate marker of HPV-driven transformation, although p16 immunostaining is not always consistent with HPV infection [2]. ‘True’ HPV-related OPSCC is considered both p16-positive and HPV DNA-positive (p16+/HPV+) [3]. The survival of OPSCC patients without distant metastasis at the time of diagnosis who receive definitive treatment significantly differs in accordance with the tumor p16 and HPV DNA status [4]. Patients with p16+/HPV+ OPSCC have the most favorable survival, followed by those with p16-positive but HPV DNA-negative (p16+/HPV-) OPSCC, and patients with p16-negative (p16-) OPSCC have the shortest survival irrespective of HPV DNA status. Of note, tumor HPV DNA status was also associated with the survival of non-oropharyngeal HNSCC patients without distant metastasis at the time of diagnosis [5].

HNSCC patients with distant metastasis at the time of diagnosis generally receive palliative treatment or best supportive care and their survival is poor. The impact of tumor HPV status on survival of HNSCC patients with distant metastasis at the time of diagnosis remained unknown for many decades. Very recently, Kaplon et al. were the first and only to report that the tumor HPV DNA status correlates with the survival of OPSCC patients with distant metastasis at the time of diagnosis [6]. They queried the National Cancer Database (NCDB) for OPSCC and analyzed survival in terms of tumor HPV DNA status. They found that tumor HPV DNA status did not have a significant impact on the survival of patients receiving no treatment, while HPV DNA positivity favored survival in patients receiving any kind of treatment. The two-year overall survival of HPV+ OPSCC patients was 42%, although 7% of patients received no treatment. The median overall survival of HPV+ OPSCC patients receiving treatments, such as chemotherapy alone, radiotherapy alone, or chemotherapy plus radiotherapy, was 20 months. However, it remains unclear whether patients received treatments in a definitive or palliative setting because the NCDB does not capture the specific information regarding treatment regimens. Moreover, p16-/HPV+ OPSCC patients might have been included in the analysis. Collectively, the prognosis of ‘true’ HPV-related HNSCC patients with distant metastasis at the time of diagnosis who receive definitive treatment remains unknown.

Dying tumor cells release small DNA fragments into the bloodstream, which represents circulating tumor DNA (ctDNA) [7]. ctDNA offers a non-invasive approach to monitor therapeutic efficacy in real time. E6 or E7 HPV DNA in plasma serves as ctDNA in HPV-related HNSCC [8,9]. Circulating tumor HPV DNA (ctHPVDNA) levels decreased after induction chemotherapy compared to its pre-treatment levels [10]. However, the kinetics of ctHPVDNA during induction chemotherapy and its association with disease control remains unknown.

In the setting of daily clinical practice at our institution since 2013, HNSCC patients with distant metastasis at the time of diagnosis initially undergo platinum-based triplet induction chemotherapy if they are tolerable to and willing to receive it. Subsequently, they are encouraged to undergo radiotherapy for locoregional disease if their distant diseases are adequately responsive to induction chemotherapy. Furthermore, since June 2017, we have been serially collecting blood samples from HPV-related HNSCC patients at baseline and during and after treatment and have been monitoring ctHPVDNA. Herein, we examined the survival of HNSCC patients with distant metastasis at the time of diagnosis who received platinum-based triplet induction chemotherapy in an attempt to reveal the impact of tumor HPV status on survival. We also analyzed the kinetics of ctHPVDNA in the context of response to induction chemotherapy in a subset of patients. Molecular response based on ctHPVDNA and radiographic response based on computed tomography (CT) or 18F-fluorodeoxyglucose positron emission tomography/CT (FDG-PET/CT) were compared.

## Materials and methods

Patients and treatment

**Figure 1:**
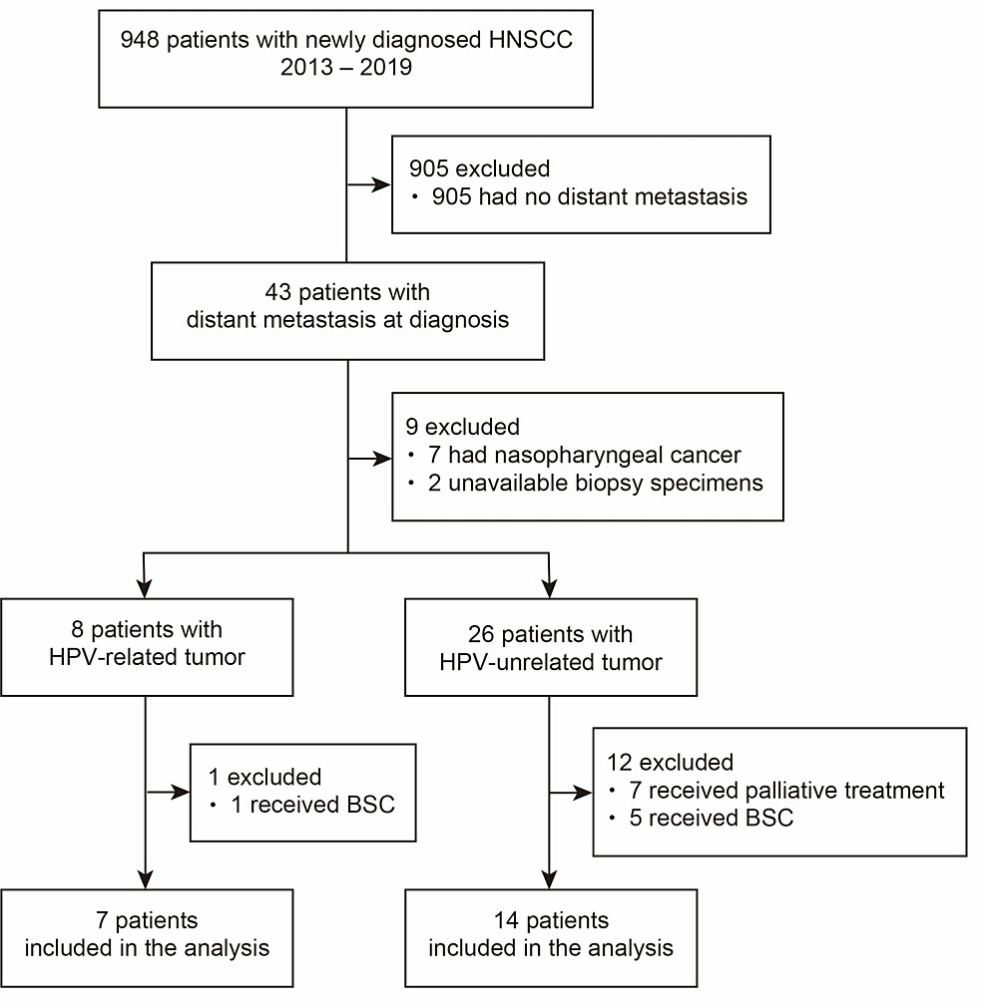
Schematic representation of the method of patient identification. BSC, best supportive care; HNSCC squamous cell carcinoma of the head and neck; HPV, human papillomavirus.

As shown in Figure 1, we performed a medical chart review for 948 patients with newly diagnosed HNSCC, who first visited Osaka University Hospital between 2013 and 2019. We identified 21 patients with distant metastasis who received platinum-based triplet induction chemotherapy. A triplet regimen was either modified TPF or modified TPEx. The dosage of chemotherapeutic reagents was reduced by 20% from the original regimens [11,12]. The modified TPF regimen comprised docetaxel 60 mg/m^2^ on day 1, cisplatin 60 mg/m^2^ on day 1, and fluorouracil 600 mg/m^2^ on days 1-5 and the modified TPEx regimen comprised docetaxel 60 mg/m^2^ on day 1, cisplatin 60 mg/m^2^ on day 1, and cetuximab on days 1, 8, and 15 (400 mg/m^2^ day 1 of the first cycle and 250 mg/m^2^ weekly on subsequent administrations). A total of three and four cycles were prescribed for TPF and TPEx, respectively, and repeated every 21 days. All patients received CT and FDG-PET/CT at baseline and CT after the completion of induction chemotherapy. Some patients received CT after the second cycle of induction chemotherapy for interim assessment of anatomic response, and some patients received FDG-PET/CT after the completion of induction chemotherapy for assessment of metabolic response. Overall end-of-induction chemotherapy anatomic response was assessed in accordance with the Response Evaluation Criteria in Solid Tumors version 1.1 [13]. Metabolic response was defined as follows: complete metabolic response (CMR) as no FDG uptake over background in locations consistent with tumors, partial metabolic response (PMR) as mild FDG uptake, stable metabolic disease as no visible change in FDG uptake, and progressive metabolic disease as increase in intensity or extent of FDG uptake or new FDG-avid lesions. The tumor HPV DNA status was evaluated through nested polymerase chain reaction (PCR) and direct sequencing of fresh frozen biopsy specimens as described previously [14]. Biopsy specimens were immunohistochemically examined for p16, using antibody clone p16 CINtec E6H4 (Roche, Basel, Switzerland). p16 was scored positive if ≥70% of tumor cells displayed robust and diffuse nuclear and cytoplasmic staining. p16+/HPV+ tumors were defined as HPV-related, while p16+/HPV- or p16- tumors were defined as HPV-unrelated. TNM classification was based on the seventh edition of the Union for International Cancer Control TNM Classification system [15]. This study was approved by the Institutional Review Board, and patients participating in HPV DNA testing provided written informed consent.

Quantification of ctHPV16DNA

In a subset of patients, circulating tumor HPV16 DNA (ctHPV16DNA) levels were serially monitored. During induction chemotherapy, monitoring was carried out after every chemotherapy cycle. Blood samples (8.5 mL) were obtained using a Cell-Free DNA Collection Tube (Roche Diagnostics, Switzerland). Cell-free DNA was extracted from 3 mL of cryopreserved plasma using the QIAamp Circulating Nucleic Acid Kit (QIAGEN, Hilden, Germany) and was measured using the Qubit 2.0 fluorometer and the Qubit dsDNA HS Assay Kit (Invitrogen, Carlsbad, CA, USA). HPV16 E6 and E7 copy number were quantified in triplicate through optimized droplet digital PCR (ddPCR) using the QX200 Droplet Digital PCR System (Bio-Rad, Hercules, CA, USA) as we reported previously [16]. Droplet absorbance was determined using the QX200 Droplet Reader (Bio-Rad) with the threshold being 2,500 for E6 and 2,000 for E7. The results were analyzed with the QuantaSoft software v1.7.4.0917 (Bio-Rad). The number of ctHPV16DNA copies per mL of plasma was defined as the average of the number of E6 copies per mL of plasma and the number of E7 copies per mL of plasma.

Statistical analysis

Fisher’s exact test and the Wilcoxon rank-sum test were performed to compare categorical and continuous variables, respectively. Overall survival was measured from the first visit to the final visit or death. We estimated overall survival through the Kaplan-Meier method and carried out comparisons using the log-rank test. All data were updated as of June 2020. All statistical analyses were performed using JMP Pro 14 (SAS Institute, Cary, NC, USA) and P < 0.05 was considered statistically significant.

The study protocol was approved by the institutional review boards of the Osaka University Hospital (approval number 16329-3). Informed consent was waived because of the retrospective nature of this study.

## Results

Response and survival

**Figure 2:**
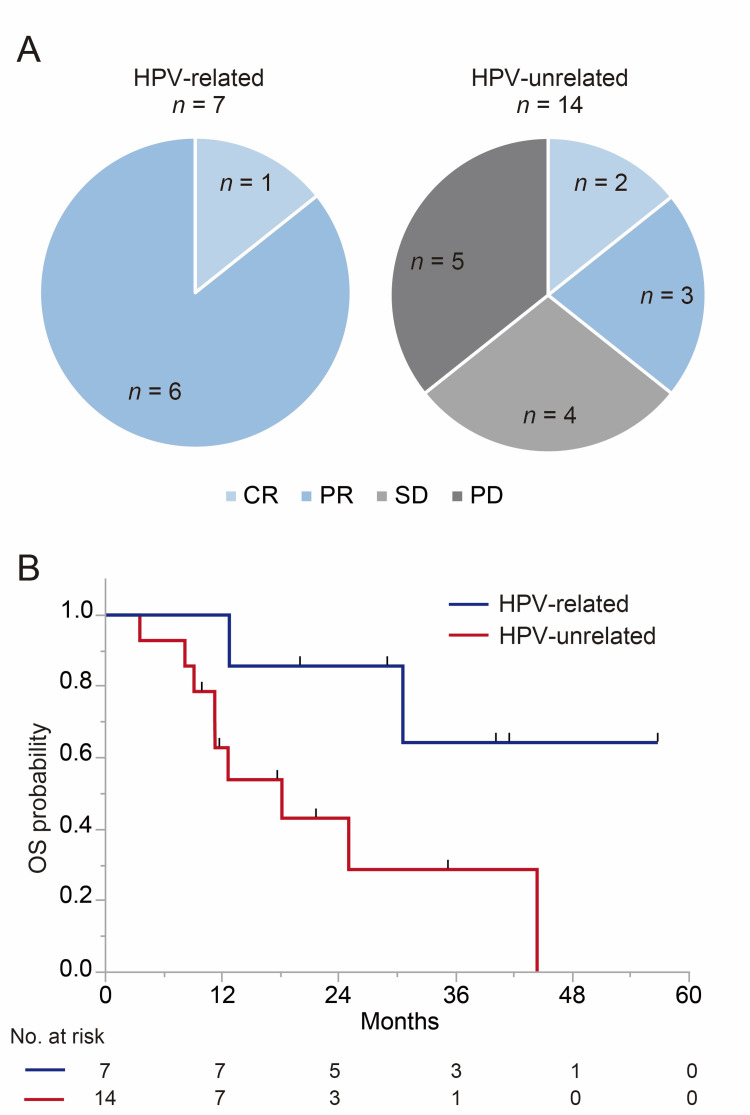
Response to induction chemotherapy and survival. (A) Overall end-of-induction chemotherapy response. (B) Kaplan-Meier estimates of overall survival. CR, complete response; HPV, human papillomavirus; PD, progressive disease; PR, partial response; SD, stable disease.

Patient baseline characteristics are summarized in Table 1. Among 21 patients receiving platinum-based triplet induction chemotherapy, seven and 14 patients had HPV-related and HPV-unrelated HNSCC, respectively. All HPV-related HNSCC patients had tumors genotyped as HPV16. A patient with an unknown primary cancer underwent open biopsy of the neck because fine needle aspiration was uninformative, which revealed the tumor to be p16+/HPV+. All seven HPV-related HNSCC patients had a complete response (CR) or partial response (PR) to induction chemotherapy, whereas five (36%) of 14 HPV-unrelated HNSCC patients had a CR or PR (P = 0.007, Figure 2A). After induction chemotherapy, all but one (86%) patients with HPV-related HNSCC received radiotherapy, while only five (36%) patients with HPV-unrelated HNSCC (P = 0.06). One patient with HPV-related HNSCC refused radiotherapy though he had a good PR to induction chemotherapy with distant lesions disappeared. Nine (64%) of 14 HPV-unrelated HNSCC patients did not proceed to radiotherapy because of a poor response to induction chemotherapy (stable disease or progressive disease). With a median follow-up of 26 months (range, 12-57 months) in surviving patients, the two-year overall survival was 86% in HPV-related HNSCC patients and 43% in HPV-unrelated HNSCC patients (P = 0.04, Figure 2B). The median overall survival of HPV-unrelated HNSCC patients was 18 months, which was not reached in HPV-related HNSCC patients. 

**Table 1:** Patient characteristics. Categorical variables are presented as number (n) and percentage (%) of patients. Continuous variables are presented as median and range. * 7th edition of UICC HPV, human papillomavirus; TPEx, docetaxel/cisplatin/cetuximab; TPF, docetaxel/cisplatin/fluorouracil; UICC, Union for International Cancer Control

	HPV-related n = 7	HPV-unrelated n = 14	
Variables	n (%)	n (%)	P-value
Age			0.60
Median	62	66	
Range	60–74	53–73	
Sex			1.00
Male	6 (86)	12 (86)	
Female	1 (14)	2 (14)	
Primary site			0.15
Oral cavity	0 (0)	1 (7)	
Oropharynx	5 (71)	5 (36)	
Hypopharynx	0 (0)	6 (43)	
Larynx	1 (14)	2 (14)	
Unknown primary	1 (14)	0 (0)	
p16 and HPV DNA status			< 0.001
p16+/HPV+	7 (100)	0 (0)	
p16+/HPV-	0 (0)	1 (7)	
p16–	0 (0)	13 (93)	
T stage*			0.66
TX–T2	3 (43)	8 (57)	
T3, T4	4 (57)	6 (43)	
N stage*			0.66
N1–N2b	2 (29)	6 (43)	
N2c, N3	5 (71)	8 (57)	
Number of metastatic lesion			1.00
1	2 (29)	4 (29)	
2–5	3 (43)	6 (43)	
6 or more	2 (29)	4 (29)	
Triplet regimen			0.66
TPF	2 (29)	6 (43)	
TPEx	5 (71)	8 (57)	
Number of cycles			0.17
1, 2	1 (14)	7 (50)	
3, 4	6 (86)	7 (50)	
Radiotherapy			0.06
Yes	6 (86)	5 (36)	
No	1 (14)	9 (64)	
Dose of radiotherapy			0.35
Median	70	70	
Range	66–70	62–70	

Kinetics of ctHPV16DNA

**Figure 3:**
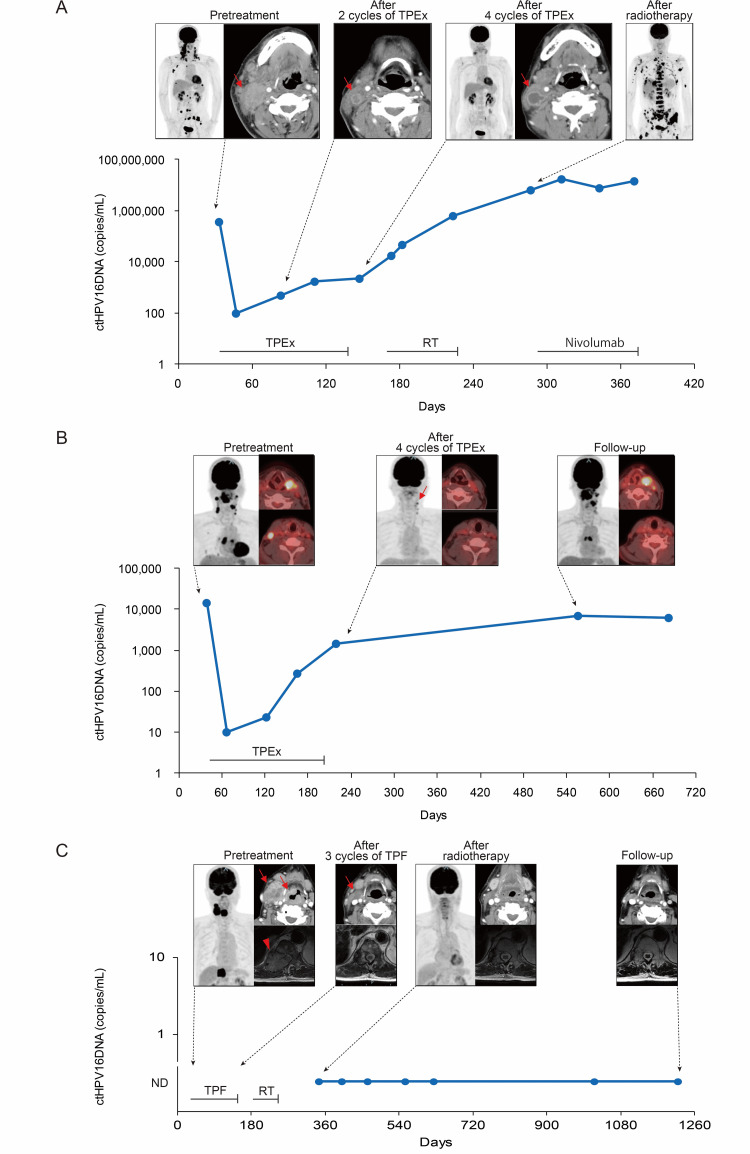
The kinetics of ctHPV16DNA and radiographic findings in metastatic HPV-related HNSCC patients receiving induction chemotherapy. (A) A 61-year-old man with cTXN2cM1 unknown primary cancer. He received four cycles of induction TPEx. Subsequently, he received radiotherapy at 70 Gy for locoregional disease. Thereafter, he received nivolumab, an anti-programmed death-1 monoclonal antibody but died of disease. The arrow indicates the neck node that shrank after the second cycle of induction TPEx, which regrew after the fourth cycle. He had a good PR and good PMR after the fourth cycle of induction TPEx but had a PMD after radiotherapy. ctHPV16DNA decreased after the first cycle of induction TPEx but thereafter continued to increase. (B) A 61-year-old man with cT4aN2cM1 oropharyngeal cancer. He received four cycles of induction TPEx and had a good PMR. He refused radiotherapy and was followed up without additional treatment. He remained alive with the disease. ctHPV16DNA decreased after the first cycle of induction TPEx but thereafter continued to increase. The arrow indicates residual nodal disease after induction TPEx. (C) A 74-year-old man with cT3N2bM1 laryngeal cancer, harboring a bulky distant metastatic lesion in the lumber vertebrate. He received three cycles of induction TPF and had a locoregional PR and distant CR. Subsequently, he received radiotherapy at 66 Gy for locoregional disease and 40 Gy for distant disease and had an overall CR and CMR. Thereafter, he remained disease-free. After radiotherapy, ctHPV16DNA remained undetectable. The arrow and arrowhead indicate locoregional and distant lesions, respectively. Day 0 is day of first visit. CMR, complete metabolic response; CR, complete response; ctHPV16DNA, circulating tumor HPV type16 DNA; HPV, human papillomavirus; ND, not detectable; PMD, progressive metabolic disease; PMR partial metabolic response; PR, partial response; TPEx, docetaxel/cisplatin/cetuximab; TPF, docetaxel/cisplatin/fluorouracil.

We monitored ctHPV16DNA in two patients throughout their clinical course. In one patient, the ctHPV16DNA level was markedly decreased after the first cycle of induction chemotherapy but then became upregulated after the second cycle and continued to increase thereafter, despite additional chemotherapy (Figure 3A). The tumor potentially became drug resistant by the end of the second cycle of induction chemotherapy. Contrast-enhanced CT (CE-CT) revealed that all metastatic neck nodes dwindled after the second cycle of induction chemotherapy. However, one neck node was enlarged after the fourth cycle, although its size remained smaller than the one recorded pre-treatment. The overall metabolic response, evaluated through FDG-PET/CT after the fourth cycle of induction chemotherapy, was excellent, notwithstanding avid FDG-uptake in the re-growing node. Radiographically, the patient had a good PR on CE-CT and a good partial metabolic response (PMR) on FDG-PET/CT. Nonetheless, the CE-CT findings corroborate the acquisition of drug resistance. In particular, the kinetics of ctHPV16DNA revealed the drug resistance after the second cycle of induction therapy, whereas CE-CT assessment revealed drug resistance only after the fourth cycle. Importantly, the acquisition of drug resistance could not have been identified radiographically without interim CE-CT assessment after the second cycle. ctHPV16DNA level further increased during radiotherapy. Thereafter, locoregional disease was almost obliterated, while distant disease exhibited striking progression. Subsequently, the patient received immunotherapy, and ctHPV16DNA levels marginally decreased. The other patient also received four cycles of induction chemotherapy, presenting similar kinetics of ctHPV16DNA and radiographic findings (Figure 3B). Radiographically, this patient had an excellent response to induction chemotherapy: a good PR on CT and good PMR on FDG-PET/CT. On the contrary, the kinetics of ctHPV16DNA suggested the establishment of drug resistance by the end of the second cycle. The patient refused radiotherapy after induction chemotherapy and was followed up without additional treatment. ctHPV16DNA levels increased with time. Follow-up FDG-PET/CT revealed that some original lesions relapsed and some remained in remission, indicating tumor heterogeneity in drug resistance. Collectively, the results of these two patients indicate that molecular response based on ctHPV16DNA precedes radiographic response, such as anatomic response based on CE-CT or metabolic response based on FDG-PET/CT, in identifying the establishment of drug resistance. ctHPV16DNA was also monitored in three other patients, though only after radiotherapy. All three patients had undetectable ctHPV16DNA levels at any time point and remained disease-free. Figure 3C depicts a representative case.

**Figure 4:**
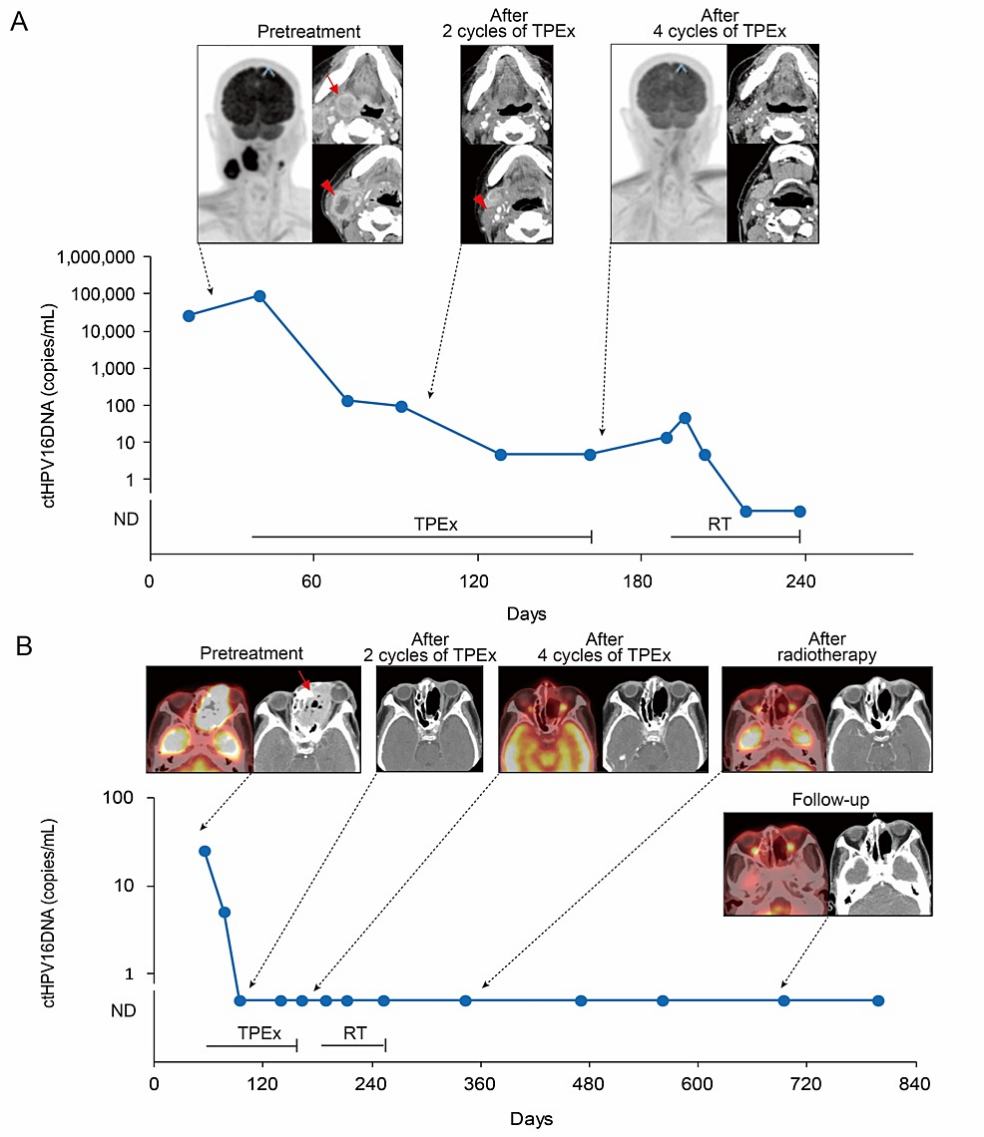
The kinetics of ctHPV16DNA and radiographic findings in non-metastatic HPV-related HNSCC patients receiving induction chemotherapy. (A) A 72-year-old man with cT3N2cM0 oropharyngeal cancer. He received four cycles of induction TPEx. He had a PR after the second cycle and a CR and CMR after the fourth cycle of induction TPEx. ctHPV16DNA declined after every cycle of induction TPEx but stayed marginally detectable after the fourth cycle. He subsequently received radiotherapy and ctHPV16DNA became undetectable during radiotherapy. The arrow and arrowhead indicate local and regional lesions, respectively. (B) A 69-year-old man with cT4bN0M0 nasal cancer. He received four cycles of induction TPEx followed by radiotherapy at 70 Gy. He had a CR after the second cycle and a CR and CMR after the fourth cycle of induction TPEx. FDG-PET/CT at three months after radiotherapy and during follow-up revealed no metabolically active disease. ctHPV16DNA became undetectable after the second cycle of induction TPEx and remained undetectable thereafter. The arrow and arrowhead indicate the primary lesion and metastatic neck node, respectively. Day 0 is day of first visit. CMR, complete metabolic response; CR, complete response; ctHPV16DNA, circulating tumor HPV type16 DNA; FDG-PET/CT; 18F-fluorodeoxyglucose positron emission tomography/computed tomography; HPV, human papillomavirus; ND, not detectable; PR, partial response; TPEx, docetaxel/cisplatin/cetuximab.

Unfortunately, blood samples were not collected from the remaining HPV-related HNSCC patients with distant metastasis at the time of diagnosis who received induction chemotherapy. Therefore, the kinetics of ctHPV16DNA during induction chemotherapy was unclear in patients who had a CR to induction chemotherapy. Instead, we show two HPV-related HNSCC patients without distant metastasis at the time of diagnosis who had a CR to induction chemotherapy (Figure 4). In one patient, the ctHPV16DNA level decreased after every cycle of induction chemotherapy but ctHPV16DNA was marginally detectable after the fourth cycle, indicating that molecular response to induction chemotherapy was incomplete. In contrast, radiographic response was complete: a CR on CE-CT and CMR on FDG-PET/CT (Figure 4A). This patient proceeded to receive radiotherapy. In the meantime, the ctHPV16DNA level increased and then became undetectable during radiotherapy, suggesting that subclinical active disease that was radiographically unidentifiable persisted after induction chemotherapy. In the other patient, ctHPV16DNA became undetectable after the second cycle of induction chemotherapy and thereafter remained undetectable, indicating that molecular response to induction chemotherapy was complete. Radiographic response to induction chemotherapy was also complete: a CR on CE-CT and CMR on FDG-PET/CT (Figure 4B). Subsequently, this patient received radiotherapy and remained disease-free during follow-up. These results indicate that there is a discordance between molecular and radiographic responses to induction chemotherapy and that ctHPV16DNA is detectable in the presence of active disease irrespective of radiographic findings. ctHPV16DNA is a more precise predictor of response to induction chemotherapy than radiographic images.

## Discussion

We demonstrated for the first time that “true” HPV-related HNSCC patients with distant metastasis at the time of diagnosis had a favorable prognosis after treatment with platinum-based triplet induction chemotherapy followed by radiotherapy. It is suggested that definitive treatment rather than palliative treatment or best supportive care should be considered for these patients. We used two platinum-based triplet regimens of TPF and TPEx for induction chemotherapy; however, the optimal regimen remains unknown. Recently, pembrolizumab along with platinum and fluorouracil is administered as first-line therapy for recurrent and/or metastatic HNSCC [17]. It would be of interest to determine the effectiveness of this new triplet regimen as induction chemotherapy.

Vermorken et al. retrospectively assessed recurrent and/or metastatic HNSCC patients treated with platinum-based chemotherapy with or without cetuximab and reported that p16+ and HPV+ HNSCC patients survived longer than p16- and HPV- HNSCC patients, respectively, irrespective of cetuximab status, although the differences were not significant [18]. The median overall survival was the longest (13 months) among HPV+ HNSCC patients receiving chemotherapy and cetuximab. Furthermore, the response rate to treatment was better for p16+ and HPV+ HNSCC patients than for p16- and HPV- HNSCC patients, respectively. The response rate peaked at 64% for HPV+ HNSCC patients receiving chemotherapy and cetuximab. Given our finding that the response rate was 100% and median overall survival was not reached in p16+/HPV+ HNSCC patients, recurrent HNSCC might poorly respond to treatment compared to metastatic HNSCC at the time of diagnosis. This difference in treatment responses is probably due to private 'driver' mutations. Hu et al. reported that post-treatment metastases are often characterized by the presence of private 'driver' mutations associated with resistance to chemotherapeutic agents, which is not the case in treatment-naïve metastasis [19].

We also demonstrated for the first time that monitoring of ctHPVDNA during induction chemotherapy successfully identified the establishment of drug resistance earlier than radiographic assessment. Considering that ctHPVDNA levels continuously increased after the second cycle of induction chemotherapy, the third and fourth cycles of induction chemotherapy were clearly useless in disease control. From a perspective of disease control, it would be better to switch promptly to the second-line chemotherapy with radiotherapy suspended rather than continue the first-line chemotherapy once monitoring of ctHPVDNA suggests the establishment of drug resistance to the first-line chemotherapy. Further studies are required to resolve this issue.

Undetectable ctHPVDNA during induction chemotherapy might represent the absence of active residual disease. In other words, continuous chemotherapy after ctHPVDNA becoming undetectable might be excessive. Patients in whom ctHPVDNA becomes undetectable during induction chemotherapy might be allowed to skip to subsequent radiotherapy without accomplishing prescribed cycles of chemotherapy, which might not compromise survival. This is an issue of clinically great interest, which should be addressed in future trials. It is important to note that numerous clinical trials assessing the utility of de-intensified treatment in HPV-related OPSCC have been or are being studied, for which the treatment regimen often includes three cycles of induction chemotherapy followed by radiotherapy [20-22]. Moreover, interim radiographic assessment of chemotherapeutic responses is generally not scheduled. In order to achieve ‘true’ treatment de-intensification, monitoring of ctHPVDNA might serve to evade the continuation of potentially excessive chemotherapy.

There are a series of reports showing the utility of ctHPVDNA in the management of HPV-related HNSCC. The clearance profile of ctHPVDNA during concurrent chemoradiotherapy was associated with disease control [23]. The detection of ctHPVDNA at three months after (chemo)radiotherapy predicted residual or recurrent disease accurately [16]. The detection of ctHPVDNA during post-treatment surveillance preceded the radiographic diagnosis of disease recurrence [24]. Now we have added a new value to the utility of ctHPVDNA: monitoring of ctHPVDNA during induction chemotherapy could help the decision-making of the continuation or discontinuation of chemotherapy. ctHPVDNA is a promising less-invasive biomarker that is highly useful in the management of HPV-related HNSCC.

The primary study limitation was that this was a retrospective study with a small patient cohort; hence, further prospective studies with larger cohorts are required to investigate the effectiveness of platinum-based triplet induction chemotherapy followed by radiotherapy among HPV-related HNSCC patients with distant metastasis at the time of diagnosis.

## Conclusions

The survival of HPV-related HNSCC patients with distant metastasis at the time of diagnosis is favorable after treatment with platinum-based triplet induction chemotherapy followed by radiotherapy. HPV-related HNSCC patients should be treated with curative intent even in the presence of distant metastasis at the time of diagnosis. The ctHPVDNA level reflects real-time disease activity and ctHPVDNA-guided molecular response assessment precedes radiographic response assessment in identifying the acquisition of drug resistance by tumors. Monitoring ctHPVDNA during induction chemotherapy could help the decision-making of the continuation or discontinuation of chemotherapy. Further studies are warranted.
